# Digital Conversational Agents for the Mental Health of Treatment-Seeking Youth: Scoping Review

**DOI:** 10.2196/77098

**Published:** 2025-11-07

**Authors:** Lisa D Hawke, Jingyi Hou, Anh T P Nguyen, Thalia Phi, Jamie Gibson, Brian Ritchie, Gillian Strudwick, Terri Rodak, Louise Gallagher

**Affiliations:** 1 Centre for Addiction and Mental Health Toronto Canada; 2 Kama.AI Toronto, ON Canada

**Keywords:** chatbot, artificial intelligence, youth, mental health, substance use

## Abstract

**Background:**

Digital conversational agents (or “chatbots”) that can generate human-like conversations have recently been adapted as a means of administering mental health interventions. However, their development for youth seeking mental health services requires further investigation.

**Objective:**

This youth-engaged scoping review synthesizes the recent research on digital conversational agents for youth seeking mental health or substance use services.

**Methods:**

Studies were included if they were published between 2016 and 2025 and examined digital conversational agents for youth aged 11 to 24 years with mental health or substance use challenges in clinical settings. Systematic literature searches were conducted in February 2024 in multiple databases and updated in March 2025. Data were extracted using codeveloped forms and synthesized narratively.

**Results:**

Ten studies were included, all focusing on mental health. Seven examined the acceptability and feasibility of digital conversational agents; others explored youth perceptions of use, design, and content, with some exploration of impact on mental health symptoms. Eight of ten studies reported high acceptability or positive user experiences. Three were randomized controlled trials that found potential reductions in depressive symptoms. Reporting on the ethical standards was limited. No studies focused on substance use alone.

**Conclusions:**

Literature on digital conversational agents for treatment-seeking youth is emerging but limited. Future rigorous research is needed that prioritizes data security, safety measures, and youth co-design in the development of safe, engaging, digital conversational agents for youth with mental health conditions.

## Introduction

### Background

Digital conversational agents, or “chatbots,” are tools that can simulate a human interaction in text format, using artificial intelligence to respond intelligently to user inputs. They may use human-generated algorithms to produce conversations, or natural language processing to recognize and respond to inputs with a programmed personality. Digital conversational agents have been used in mental health for tasks such as providing psychoeducation, building coping skills, delivering cognitive-behavioral therapy, and providing diagnostic support [1-4].

There is emerging research available on digital conversational agents for mental health or substance use in treatment-seeking populations such as youth who are seeking or accessing services to support their mental health or substance use. A recent set of 2 reviews found minimal research on digital conversational agents for mental illness, with a specific gap around youth, and the studies were not necessarily in treatment-seeking populations [3,4]. They did find some acceptability and positive effects for the tool’s ability to establish diagnosis, but less so among youth. Some positive mental health impacts were found, but study limitations made these difficult to determine. Digital conversational agents in the psychiatric sphere have targeted various outcomes. For example, one was designed to promote activity engagement among individuals with severe mental illness [5]. Others have been designed to promote service utilization [6], develop mindfulness and emotion regulation [7], determine diagnoses [8], or promote medication adherence [9]. Evidence of the development of something akin to a therapeutic alliance has been demonstrated [10], while the responses of digital conversational agents have been rated as higher quality and more empathetic than physician responses [11]. However, the research evidence for treatment-seeking youth in clinical contexts (ie, receiving treatment) remains limited and has yet to be synthesized.

There is more research on the use of digital conversational agents as a prevention, promotion, or low-threshold mental health support tool, particularly for youth in school settings [12-15]. An acceptability study found that youth preferred to receive information through a digital conversational agent versus searching for it online, while the “personality” of the digital conversational agent was appreciated for its friendly, relational aspects [12]; the practical advice and referrals offered were also appreciated. Advantages include flexibility, accessibility, the capacity to reach more youth faster than in-person services, cost-effectiveness, and the ability to get help without stigma [12,13,15]. However, evidence on efficacy remains limited [16], and the need to empirically test these interventions has been highlighted [12,15,17].

As digital conversational agents expand around the world, a number of ethical issues have been raised. One team set out recommendations for minimal ethical standards in the use of digital conversational agents for youth mental health [17]. Some factors include ensuring confidentiality, being transparent about uses and limits, generating appropriate crisis responses, and protecting against addictive use. The article further highlighted the importance of testing efficacy, being transparent with users about the evidence base, encouraging in-person services, and explaining that these tools are not designed to treat severe mental illness. For vulnerable youth in treatment-seeking contexts, privacy and confidentiality is important, and addictive use might be a particular concern. These concerns have been reflected in discussions of the promise and necessary cautions regarding the implementation of digital conversational agents [18,19]. These issues must be attended to in the development, testing, and scale of digital conversational agents for mental health.

Youth are high users of digital technology [20] and often use digital means of seeking information about health [21]. They may be interested in receiving support through digital conversational agent technology. However, treatment-seeking youth with potentially complex mental health challenges have different needs and may have different preferences than school-based samples. Given ethical concerns about digital conversational agents for severe mental illness [17], further exploratory work is an essential prerequisite to digital conversational agent development. It is important to gain the perspectives of treatment-seeking youth around the possible utility, functionality, acceptability, and safety of digital conversational agents to address mental health challenges among them. It is also important to continue to do up-to-date work in this area, given the fast pace of technology development that leaves older research quickly out of date.

### Objective

This scoping review aims to synthesize the recent literature on digital conversational agents for youth in clinical contexts seeking treatment for mental health or substance use challenges.

## Methods

### Overview

We used the scoping review methodology given the breadth and nature of the research and the exploratory nature of the research question in an emerging field, using established scoping review processes [22,23]. The scoping review incorporated the following major steps: (1) defining the research question, (2) identifying relevant studies, (3) screening and selecting studies, (4) extracting the data, and (5) summarizing the data and reporting. PRISMA-ScR (Preferred Reporting Items for Systematic Reviews and Meta-Analyses Extension for Scoping Reviews) guidelines have been followed in the reporting of this review (Multimedia Appendix 1) [24].

### Youth Engagement

In accordance with the strategy for patient-oriented research [25], we engaged 2 youth engagement specialists with lived experience in the conduct of this review. We endorsed pragmatism as a fundamental worldview and valued youth contributions, as per lived experience engagement paradigms [26]. Youth engagement specialists helped identify keywords for the systematic search, coselected information to extract from the resulting studies, and assisted with interpreting and reporting on the findings. Collaboration with the youth engagement specialists helped guard against bias and ensured that the study was relevant to the real-world experiences of young people, enhancing rigor.

### Defining the Research Question

This scoping review aims to understand the published academic literature on digital conversational agents for treatment-seeking youth with mental health or substance use challenges, in clinical contexts. Based on the PICO (population, intervention, comparison and outcomes) framework [27], this review focuses on youth with mental health or substance use challenges (population) and applications of digital conversational agents in treatment-seeking in clinical settings (intervention). Note that the treatment-seeking and clinical settings context was a key inclusion criterion that makes this review very specific to clinical contexts, which is a gap in the literature. It includes studies with or without comparison groups and reports on the full range of findings, regarding applications, acceptability, and outcomes related to digital conversational agents for the target population, as well as other findings highlighted in the selected literature. Literature on preventive interventions and nonclinical settings was excluded.

### Identifying Relevant Studies

A comprehensive search strategy was developed with a health sciences librarian (TR). Considering the subject areas of the research question, the search was developed, tested, and finalized in Medline, then translated and run on February 22, 2024 in the following bibliographic databases: APA PsycInfo, Medline, Embase, Cumulative Index to Nursing and Allied Health Literature (CINAHL), Web of Science, Applied Social Sciences Index and Abstracts (ASSIA), and Cochrane Central Register of Controlled Trials (CENTRAL). The search was repeated and updated on March 18, 2025. The search strategies used database-specific subject headings and keywords in natural language, as well as advanced search operators such as truncation and adjacency operators, to capture 3 main concepts: youth, digital conversational agent, and mental health or substance use. The review was limited to articles published in the past 8 years (2016-2025) due to the rapid advancement of AI technology. There were no limitations placed on language or study type. Conference abstracts were removed through the search strategy when allowed by a database, but were otherwise included. A supplementary hand search of reference lists and Google Scholar was conducted. Given the nature of the research question, the gray literature search was limited to dissertations in APA PsycInfo, registered clinical trials in the CENTRAL search, and nontraditional publications indexed in CINAHL.

### Study Screening and Selection

To be included, articles had to present descriptive information or research results on digital conversational agents for youth mental health or substance use, focusing on treatment-seeking populations in clinical contexts. Articles could report on any study design and sample size and could include descriptive process papers. The digital agent and study had to be focused on youth approximately aged 11-24 years, or separate analyses had to be available for a subgroup within this age range. Articles had to be published in English or French consistent with the language skills available on the team, although no non-English language texts were found. We included any outcomes identified by the article in question, including acceptability and feasibility outcomes as well as mental health or substance use outcomes of any kind or intensity. Excluded were any studies not reporting on digital conversational agents for youth mental health or substance use, research conducted on children (majority aged younger than 11 years) or adults (majority aged older than 24 years), and commentaries that did not describe either research results or a specific digital conversational agent. Textbox 1 presents inclusion and exclusion criteria, which were developed to optimize the ability to address the research question. Selected articles were screened first at the title and abstract level by 2 of 3 research staff (include or exclude responses), with any conflicts resolved through discussion with the project lead. The resulting set of articles were screened in full using the same process. Article screening was conducted using Covidence software [28].

**Inclusion and exclusion criteria for literature search.**

Inclusion criteria

- Addresses digital conversational agents
- Agent designed for youth aged 11-24 years
- Participants with mental health or substance use challenges (disorders or symptoms)
- Participants seeking mental health or substance use treatment (ie, connected with clinical contexts)
- Published between 2016 and 2025
- Reports on efficacy, effectiveness, feasibility, or acceptability
- Reports on youth perspectives using youth-reported data (any method)

Exclusion criteria

- Not a youth-focused agent and study
- Prevention or promotion that does not support known mental health or substance use challenges
- Youth were not treatment-seeking or were not exposed to the digital conversational agent in a clinical context
- Published before 2016
- Papers without original data

### Data Extraction

A data extraction form was developed collaboratively among team members, including youth. From the final selected articles, data were extracted by 1 staff member and verified by a second staff member using a spreadsheet. The data extraction form included (1) study general information (eg, reference, location, and data collection years), (2) digital conversational agent description (eg, goal or objective, application and content, context of use, development and testing, youth engagement, software and data strategy, feedback learning integration, delivery method, financial investment, privacy, content monitoring, and sustainability plan), (3) study methods (eg, study objective, design, sampling method, sample size, sample description, exclusion criteria, measures, and other treatments), (4) outcomes (eg, finding overview, youth perspectives, digital conversational agent benefits and limitations, adverse events, and study limitations), (eg, 5) ethical issues (privacy and confidentiality, efficacy, safety, and mitigating strategies), and (eg, 6) other information (author conclusions and recommendations).

### Data Summarization and Reporting

The data were summarized narratively and in table format. Quality assessment was not conducted, as this is not a priority in scoping reviews in which a wide range of diverse research is mapped.

### Ethical Considerations

Since this is a scoping review of the literature, ethics board approval was not required. There are no human participants in this article and informed consent is not required.

## Results

### Study Selection

**Figure 1:**
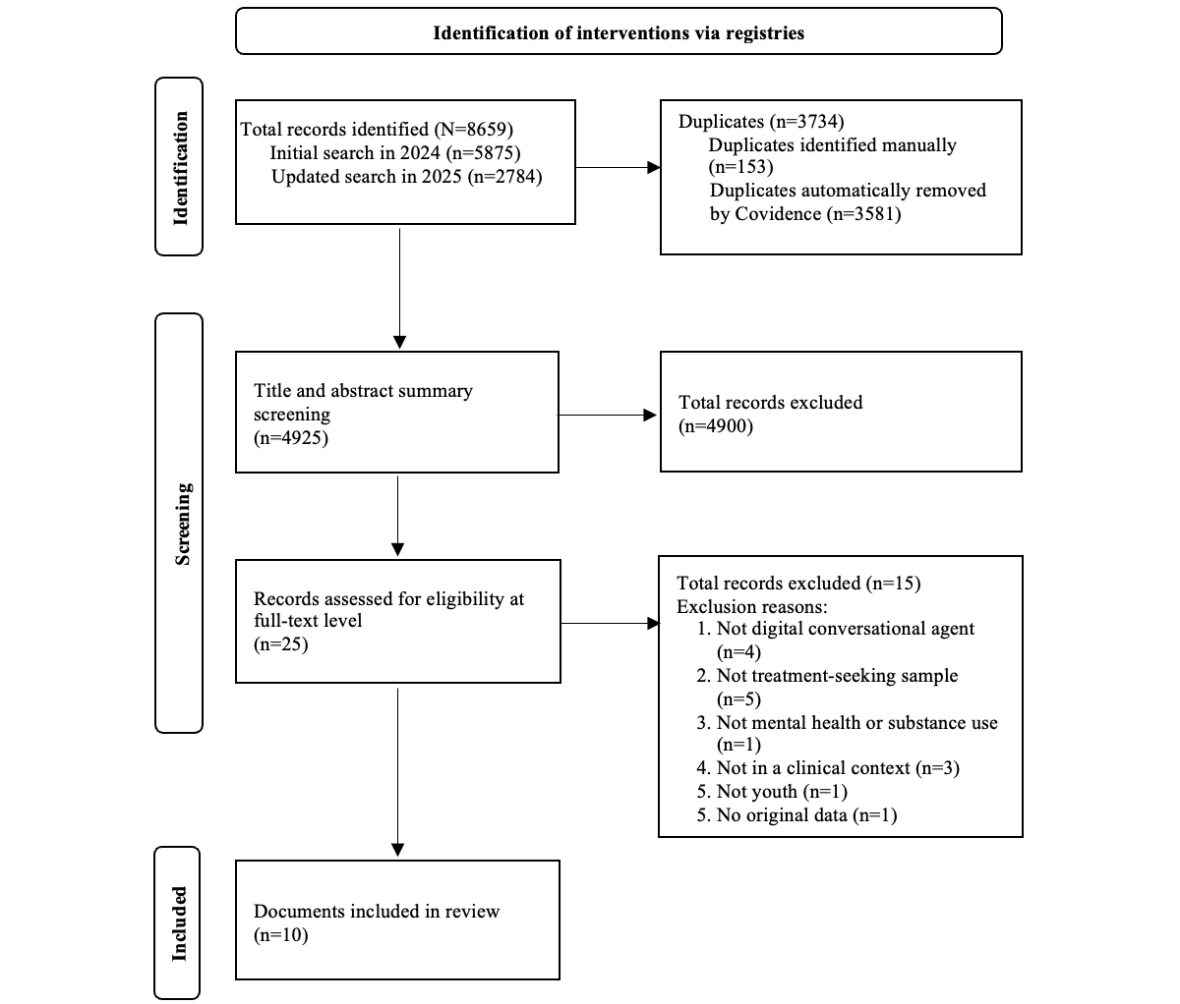
PRISMA (Preferred Reporting Items for Systematic Reviews and Meta-Analyses) flowchart.

Our search identified 8659 records, including 5875 records from the initial search in 2024 and 2784 from the updated search in 2025. After removing 3734 duplicates, we screened 4925 titles and abstracts, excluding 4900. We further assessed 25 full-text articles. Fifteen were excluded due to not meeting our inclusion criteria, which resulted in 10 studies included in the review. The study selection process is illustrated in the PRISMA flow diagram (Figure 1).

Table 1 summarizes the included articles and digital conversational agents studied. The 10 studies examined 9 digital conversational agents for youth mental health in clinical care contexts. Studies emerged from the United States [2,29,30], Germany [31,32], Australia [1,33], Thailand [34], Japan [35], and Hong Kong [36], with publication years ranging from 2017 to 2025. Only 3 articles stipulated that the digital conversational agents were co-designed with youth [1,31,33].

**Table 1:** Summary of the included articles and digital tools.

Author (year)	Country	Format	Technology described	Digital conversational agent objective	Intervention modality	Expressed digital conversational agent limitations	Codevelopment with youth
Wrightson-Hester et al [33]	Australia	Web application	Artificial intelligence conversational agent	Help youth manage mental health using curious questioning	Method of levels therapy	Repetitive content and input comprehension issues	Yes
Greenwood et al [37]	United States	Mobile app	Natural language processing and machine learning	Help youth develop emotion regulation skills	CBT, IPT-A^b^, and DBT^c^	Effectiveness not rigorously tested for; not implemented in clinical practice	Not specified
Beilharz et al [1]	Australia	Web-based chat accessible on mobile and desktop devices	Artificial intelligence conversational agent	Provide support for body image issues and eating disorders	Psychoeducation, coping skills via CBT, acceptance and commitment therapy, mindfulness	Limited to psychoeducation and simultaneous coping skills	Yes
Fitzpatrick et al [2]	United States	Instant messenger app on mobile or desktop	Decision tree methodology with predefined responses, including natural language processing at certain points within the tree	Deliver self-help CBT to young adults with anxiety and depression symptoms.	CBT	Potential risk of violating psychotherapeutic processes related to limitations in conversational abilities	Not specified
Robinson et al [30]	United States	Mobile app	Not specified	Deliver CBT to adolescents at adolescent outpatient mental health clinic.	CBT	Not specified	Not specified
Mahmud and Porntrakoon [34]	Thailand	Not specified	AI^d^-generated or DCA^e^ mental health application	No specific digital conversational agent introduced	Not specified	Lack of customization, depersonalization, algorithm bias, privacy concerns	N/A^f^
Kuhlmeier et al [31]	Germany	Mobile apps and web platforms	Text-based conversational agent prototype	Provide youth and young adults personalized therapy for depression	CBT, IPT^g^	Currently an early-stage prototype	Yes
Kuhlmeier et al [32]	Germany	Prototype accessed via laptop	Text-based conversational agent prototype	Support youth with depression through a behavioral activation exercise	Behavioral activation	Currently an early-stage prototype	No, but participant feedback will influence the future design
Hasei et al [35]	Japan	Accessible via mobile app LINE	GPT-4 large language model	Help pediatric, youth, and young adult cancer patient reduce anxiety and stress	Not specified	4-turn conversations	Not specified
Li et al [36]	Hong Kong	Accessible via Microsoft Teams	An interactive conversational system with natural language understanding	Support university students with self-reported depression through MBSR^h^	MBSR interventions	Technical issues with Microsoft Teams, required high self-discipline, limited interactivity	Not specified

^a^CBT: cognitive behavioral therapy.

^b^IPT-A: interpersonal psychotherapy for adolescents.

^c^DBT: dialectical behavior therapy.

^d^AI: artificial intelligence.

^e^DCA: digital conversational agent.

^f^N/A: not applicable.

^g^IPT: interpersonal therapy.

^h^MBSR: mindfulness-based stress reduction.

### Study Characteristics

Of the 10 included studies, 7 assessed feasibility or usability or the preliminary efficacy or effectiveness [1,2,29,33,35,36], while others investigated perceptions of using the agent [34], perceptions of their design [31], and youth’s interaction experience with them [32]. Three studies used qualitative methods [1,31,32], 1 used quantitative methods [30], and 6 used mixed methods [2,29,33-36]. Three studies used randomized controlled trials to evaluate 2 digital conversational agents [2,29,30]. Participant demographics varied in the studies, covering age groups from adolescents to young adults with depression [31,32,36], anxiety or depression [2,29,30,33,34], anxiety and stress [35], and eating disorders [1]. None focused on substance use disorders. Some studies also included mental health professionals [31,34] and caregivers [1] as participants.

### Intervention Characteristics

The technology driving the digital conversational agent was not always specified but included artificial intelligence, for example natural language processing, machine learning, and decision trees. While publications were dated 2016 to 2025, the year of the development of the digital conversational agent under examination was not reported. The interventions were delivered via a mobile app [2,29-31,35,36] or a computer browser [1,31-33]. Treatment modalities included cognitive behavioral therapy or components thereof [1,2,29-32]; an integration of cognitive behavioral therapy, interpersonal psychotherapy, and dialectical behavior therapy [29]; a method-of-levels approach [33]; mindfulness-based stress reduction interventions [36]; and unspecified modalities [34,35]. Some studies included screenshots or sample interactions to illustrate the digital conversational agent’s responses [1,31-33,35]. For example, one study shows a screenshot of coping skill recommendations made by the chatbot [1]. Another shows a screenshot of behavioral activation recommendations [32]. However, others did not provide details about the exact nature of the responses of the digital conversational agent. The digital conversational agents were not generally described as being integrated into a system of care to accompany clinical services, except for one that was designed for young cancer patients [35].

### Qualitative and Acceptability Findings

The methodology and outcomes of the included articles are summarized in Table 2. Most studies reported that the digital conversational agents were acceptable to youth, with high utilization [2,29], high satisfaction [2], and overall positive perceptions [1,2,31-33]. Participants appreciated the ease of use [33], appealing and accessible design [1,32], and friendly characters [1,32]. They also found that the content was helpful and appropriate [1,2,35], equipped them with new mental health insight [2], and offered them empathy [2,32]. Youth identified advantages, namely the neutrality, anonymity, and availability of these tools compared to traditional psychotherapy [31,35,36]; some youth indicated their willingness to use digital conversational agents for depression and anxiety, with cautions regarding privacy and security [34].

**Table 2:** Research methodology aspects of the included articles.

Author (year)	Study objective	Sample size	Sample age (years)	Mental health or substance use conditions	Exclusion criteria	Measurement	Amount of use of digital conversational agent within study	Outcome summary
Wrightson-Hester et al [33]	Assess feasibility and acceptability	13	16-24	Anxiety or depression	Severe depressive symptoms or frequent suicidal thoughts	Self-report measures of depression, anxiety, QoL^a^, related psychological constructs, and system usability and engagement Interview/focus group guide	Week 1: 1-4 days, 1-3 chats/day (average length 2-30 min); Week 2: 1-7 days, 1-5 chats/day (average length 5-15 min)	Feasible assessments; acceptable system engagement; acceptable interface; acceptable therapeutic conversations; some frustration, but resolved through use; recommendations for improvement; possible improvements in some psychological measures
Greenwood et al [37]	Establish the feasibility, preliminary acceptability, effectiveness, and usability	18 (17 analyzed)	13-17	Depression and anxiety	Parent-reported history of severe depression, substance use, psychotic illness, OCD^b^, PTSD^c^, panic disorder, or specific phobias; psychiatric hospitalization in the previous month; unaccompanied by a guardian at diagnostic visit; no access to a mobile device for regular use; unable to read and write English.	Self-report measures of depression (primary outcome), anxiety, and self-efficacy; app usage rates; acceptability, feasibility, and usability; safety monitoring.	Average use of 6 days, 55 mood check-ins, 313.17 sent messages, 13.63 lessons, and 9 tools over 4 weeks	Acceptability and feasibility confirmed; decreased depression and anxiety; increased self-efficacy; satisfactory usage rates; acceptable safety monitoring.
Beilharz et al [1]	Evaluate the acceptability and feasibility	25 (17 youth, 8 caregivers)	Youth: 13-18; caregivers: 46-57	Body image concerns or eating disorders	Not specified	Focus group guide	Not specified	Appealing character and design; preference for brief content with a conversational tone; acceptable flow via button-based navigation; preference for free-text option; appreciation of accessibility.
Fitzpatrick et al [2]	Assess feasibility, acceptability, and preliminary efficacy	70	18-28	Anxiety and depression	Not specified	Self-report measures of depression, anxiety, affect, and acceptability and usability; open-ended questions; usage tracking	Up to 20 sessions, average 12.14 check-ins over 2 weeks	Feasibility confirmed; reduction in depression symptoms, but not anxiety or positive or negative affect; high engagement and satisfaction; some positive comments on process and content; some challenges with process, technology, and content; limitations existed.
Robinson et al [30]	Explore the feasibility and noninferiority	141	13-17	Depression or anxiety	Not specified	Self-report measure of depression and anxiety	Not specified	Noninferiority to clinician-delivered CBT^d^-skills group in reducing depression symptoms.
Mahmud and Porntrakoon [34]	Explore perspectives on DCAs^e^ as alternative treatments	15 (10 youth, 5 psychologists)	Youth: 20-25	Depression or anxiety	Not specified	Interview guide; self-report measures of depression and anxiety.	Not specified	Interest in using DCA when experiencing depression or anxiety; concerns about privacy, confidentiality, and security; preferences varied on the extent and time of expert assistance
Kuhlmeier et al [31]	Design a personalized conversational agent to treat youth depression	Problem awareness phase: 15 youth; evaluation phase: 5 experts, 5 potential users	Problem awareness phase: 14-17; evaluation phase: experts: mean 29; potential users: mean 24	Depression	Not specified	Interview guide	Not specified	Personalization of the conversational agent and content is essential, combined with structure; areas of improvement identified
Kuhlmeier et al [32]	Investigate how youth interact with the conversational agent for depression	15	14-17	Depression	Suicidal ideation, psychosis, or low cognitive functioning.	User interaction data and think-aloud sessions documenting thoughts about the DCA	11:54-20:38 min	Engaging interaction style; preferences varied on emoji and language use; appreciation of predefined responses, optional reminders, and clear interface; varied perceptions of responding speed and the dialogue content
Hasei et al [35]	Assess feasibility and potential impact in pediatric, youth, and young adult cancer patients	5	13 to 20s	Not specified, but cancer patients usually experience anxiety and stress from cancer	Not specified	Self-report measure of anxiety and stress; open-ended questions.	Most used every 2-3 days for about 10-15 min/session over 1-2 weeks	Reduced anxiety and stress; possible increased cancer treatment engagement; possible increased self-expression and informal help-seeking.
Li et al [36]	Evaluate feasibility, acceptability, safety, and preliminary efficacy	30 (27 completed)	18-25	Depression	Diagnosed with a clinical psychotic condition preintervention or were currently involved in any mindfulness-based or other psychosocial interventions.	Self-report measure of depression, stress, anxiety, and mindfulness; open-ended questions.	8 weeks; encouraged at least 1 hour of daily interaction.	Feasibility, acceptability, safety, and preliminary efficacy confirmed; limitations identified.

^a^QoL: quality of life.

^b^OCD: obsessive compulsive disorder.

^c^PTSD: posttraumatic stress disorder.

^d^CBT: cognitive behavioral therapy.

^e^DCA: digital conversational agent.

A number of problems, challenges, or barriers were identified by participants across the studies. These included limited natural conversation [2,33,35], limited customization [1,31,33,34], technical glitches [2,36], and concerns regarding the quality of the therapeutic interventions [1,31,34]

### Quantitative Findings

Six studies provided quantitative results on the digital conversational agents’ ability to improve participants’ mental health. A randomized controlled trial (RCT) of a digital conversational agent called Woebot found that, compared to a self-help e-book, the intervention significantly reduced depressive symptoms after 2 weeks, with a moderate effect size (d=0.44) [2]. In another RCT, 4 weeks of using Woebot was found to be noninferior to a standard clinician-led CBT group in reducing depression when comparing pre-post average scores and the number of participants with elevated depression; the effect size was not provided [30]. In a pilot RCT, the treatment group showed greater mean improvement in depression than the waitlist control group (d=0.98) [29]. No significance testing was reported for these trials [29,30]. A single arm study found a large effect size (d=1.26) for reducing problem-related distress and a medium effect size (d=–0.66) for improving goal conflict reorganization among participants with anxiety or depression [33]. Additionally, a single arm pretest-posttest study of a digital conversational agent delivering a mindfulness intervention reported significant and large improvements in depression, anxiety, stress, and mindfulness levels, with large effect sizes across all outcomes, at d=–1.95 for stress, d =–1.53 for depression, and d=–1.09 for anxiety [36].

### Ethical and Safety Considerations

The minimal ethical standards proposed for digital conversational agents revolve around (1) privacy and confidentiality, (2) efficacy, and (3) safety [17]. One study encompassed these requirements by providing user participants with an information package regarding privacy, efficacy, and safety issues as part of the application onboarding process [29]. Another study emphasized rigorous approval and safety measures that include anonymized and secure data handling and adherence to the Declaration of Helsinki [35]. However, most of the included articles did not discuss ethical issues in depth. While concerns over privacy and confidentiality were mentioned by many youths and stakeholders [29,31,33,34], only half of the articles included any information on their privacy policies. A case series assessing feasibility and acceptability for depression or anxiety reported that secure user identification and anonymized data collection was not conducted within the study, but would be implemented in the future [33]. One study informed participants that they would not be monitored by a counsellor [1], while another disclosed that a psychologist would monitor participants’ activities asynchronously [2].

Despite the ethical importance of ensuring efficacy, most of the articles described pilot trials examining acceptability and feasibility without adequate power to determine efficacy. Prominent limitations were small sample sizes and the lack of diversity. As a result, while many of the interventions were reportedly inspired by evidence-based therapeutic approaches [1,2,29,33,36], the delivery of the interventions via these tools has not been demonstrated efficacious or effective in real-world settings and thus requires further evaluation.

Safety issues are also worthy of note. Only 6 of the selected studies described safety features [1,2,29,33,35,36]. Among these, 3 reported that participants were informed of about the tool and given explanation of its function and capacities [1,2,29]. Only 1 study mentioned having a crisis response measure, in which the digital conversational agent could identify concerning language and confirm it with users [29]. One study emphasized that their safety measures include automated escalation systems for distress detection and professional involvement during development and testing [35]. Another study involved continuous monitoring by mental health professionals, weekly participants check-ins, and mental health support resources and referral pathways [36]. The digital conversational agents featured in these 4 trials offered additional mental health resources and provided easily accessible channels to connect users with suicide helplines or emergency services.

## Discussion

### Principal Findings

This review synthesized the recent literature on digital conversational agents for youth with mental health or substance use conditions, specifically in treatment-seeking clinical contexts. It was found that the literature is quite preliminary and is focused entirely on mental health, not substance use conditions. Yet, this is an emerging area of work that is a high priority to some research teams and health care organizations. Several of the digital conversational agents reviewed applied the principles of cognitive behavioral therapy, but this was not universal. Promising findings were identified in terms of feasibility and acceptability, together with reasonable usage rates and positive youth perspectives of acceptability. Preliminary findings suggested that digital conversational agents might be an acceptable means of delivering therapeutic content to treatment-seeking youth, with positive impacts on their mental health.

The literature was largely made up of feasibility and acceptability studies rather than efficacy trials. The digital conversational agents were not described as being embedded in intervention contexts, such as being integrated with in-person clinical care. Sample sizes were small and diversity was limited. People with different sociodemographic profiles and different clinical needs might be differentially interested in using e-health innovations [38,39], although differences across population subgroups have not been definitively established. This leaves the evidence base in a very premature state. For example, there is a lack of exploration of the impact the tools might have as an integrated component of youth mental health care among diverse youth.

All of the articles addressed mental health; none addressed substance use. It is important to consider mental health and substance use together, given the high levels of overlap and increasing trends toward treating concurrent disorders together [40]. Examining digital conversational agents for substance use challenges, or for concurrent mental health and substance use challenges, is a pressing area for future work. Investigations into suitability using different treatment modalities for specific diagnostic groups might also be worthy of consideration, given that specific symptoms may interact with whether and how one might use such a tool (eg, low motivation, paranoia, suicidal ideation, and mood instability).

This review focused on very recent literature on digital conversational agents, given the fast pace of technology. However, any article published in 2016 was likely examining a digital conversational agent developed at the latest in 2015, potentially earlier. These tools were not leveraging today’s latest technology, and the technology used was not thoroughly described. The rapid pace of technology advancement is not a new challenge, but rather one that the health care sector has been grappling with for years [41]. This leaves two problems: (1) the technology is already out of date by the time the research is complete, and (2) the evidence base that a new study leverages is out of date and may no longer be relevant by the time it is used. Ongoing rapid research and publication in this area is essential, with thorough descriptions of the technology underpinning the tool, including the year of tool development to help readers understand the state of technological advancement of the digital conversational agent in question.

In digital intervention development, co-design with the end users is critical [42]. This can be done using appropriate patient engagement frameworks [43]. Co-design helps ensure that the digital tool is appropriate for the end users and relevant to their needs. However, multiple articles reviewed did not discuss co-design, which may reduce acceptability. Future digital design teams should adhere to patient engagement principles for e-health innovation [43] as well as youth engagement principles [44]. Co-design has the potential to be a meaningful opportunity for young people to gain experience in technology development and clinical research, supporting their personal and professional growth and development [45]. In conducting co-design, teams should incorporate principles of equity, diversity, inclusion and accessibility to ensure that a diverse range of voices are included, to generate a digital conversational agent that is appropriate for a wide range of diverse youth, and to mitigate the harms that can come from biases, discrimination, and inequities. Specifically, a lack of diversity at the co-design stage could perpetuate algorithmic bias, which should be guarded against. Engagement can also be leveraged to increase digital literacy and the use of resulting tools, potentially with the support of digital navigators [46].

From our review, it was clear that the minimum ethical standards for digital conversational agents were not adequately attended to [17]. Few studies reported directly on privacy, confidentiality, and safety, and efficacy has not been definitively established. We call on developers and trialists to directly attend to the proposed ethical standards in their initiatives, proactively integrate these minimum standards into their technology and trials, integrate an ethical review stage into their initiative, and report clearly on the use of these standards. The ethics of delivering evidence-based intervention modalities via digital conversational agents should be considered—and when which modality is appropriate and for whom—since a clinician may naturally adapt their services to the individual youth, while digital tools may function differently using artificial intelligence in attempt to do the same. Matching the tool’s aim to the youth’s need, ensuring youth can withdraw at any time, and offering access to a person to talk to would all further enhance tools’ ethics and should be considered by interventionists. It should be transparent that a digital conversational agent can supplement clinical care but is not a replacement for it; integrating tools into clinical care rather than leaving them as stand-alone tools might address this challenge [47].

The use of digital conversational agents is not without risk. A recent news story highlights a case of youth suicide after the youth’s ongoing interaction with a digital conversational agent [48]. It should be noted that youth are using digital conversational agents for mental health support even if these agents are not designed for that purpose. By developing a digital conversational agent with and for youth, with the explicit purpose of supporting mental health in appropriate ways using evidence-based content, it may be possible to provide young people with a safer tool and prevent such tragedies from occurring.

While some articles discussed soliciting youth feedback as part of a study, none discussed collecting youth feedback within the digital platform. Providing opportunities to submit feedback within the platform is a potential mechanism to enable continuous improvement and incremental alignment with the needs and preferences of the users. The feedback could be directed to programming teams for programming feedback, and potentially separately to clinical teams to support clinical care as part of an integrated treatment plan. It would be important to determine means to balance the importance of privacy and confidentiality [17] with the potential for identified clinical support.

The next steps in this line of work would be to address some of the limitations, such as co-design and ethical concerns, with the ultimate goal of moving forward to full-scale trials to examine efficacy. This would have to be followed by implementation and ongoing refinement. The evaluation of complex interventions is expected to begin with iterative stages of intervention development, feasibility testing, evaluation, and implementation [49,50], and the research for this type of intervention is in the early stages of this process.

### Limitations

Strengths and limitations of this review should be kept in mind. Important partners were engaged in the review, including industry partners and youth with lived experience. These partnerships ensured that the search terms, data extraction, and interpretations are relevant to the artificial intelligence industry and youth. However, readers should note the methodological and scope limitations: the search included only an 8-year period to ensure that the findings are as relevant as possible to recent technology, but may have missed early findings. We excluded any articles focusing on prevention or promotion, which may have relevant findings. Any publications indexed after March 2025 were not captured. The search was run only in English-based databases.

### Conclusions

Emerging but preliminary literature is examining digital conversational agents for treatment-seeking youth with mental health conditions, but not substance use challenges. Digital technology already exists and will inevitably expand, making it essential that we support its expansion with rigorous research. This research must therefore continue, with attention to establishing efficacy, as well as ensuring appropriate protection of privacy and confidentiality and attention to safety and ethical concerns. While digital technology comes with challenges, it also comes with potential benefits. Co-design with youth, for youth, should be a priority. Nevertheless, promising early research justifies continuing this work with the possibility of codeveloping safe, accessible, youth-friendly, and potentially efficacious tools for youth mental health.

## Appendix

Multimedia Appendix 1 PRISMA-SCR checklist.

## Abbreviations

ASSIA: Applied Social Sciences Index and Abstracts
CENTRAL: Cochrane Central Register of Controlled Trials
CINAHL: Cumulative Index to Nursing and Allied Health Literature
PICO: population, intervention, comparison and outcomes
PRISMA-ScR: Preferred Reporting Items for Systematic Reviews and Meta-Analyses Extension for Scoping Reviews
RCT: randomized controlled trial

## Data Availability

The data underlying this project are included herein. Additional data are available upon request.
